# A Novel Nuclear-Localized Micropeptide, MP60, Promotes Hepatocellular Carcinoma Progression via the Epithelial-Mesenchymal Transition

**DOI:** 10.3390/cancers17172932

**Published:** 2025-09-07

**Authors:** Chencheng Li, Xiu Hong, Sarra Setrerrahmane, Xiaoyi Sun, Xue Zhang, Hanmei Xu

**Affiliations:** 1 Jiangsu Province Engineering Research Center of Synthetic Peptide Drug Discovery and Evaluation, China Pharmaceutical University, Nanjing 210009, China; lichencheng2@163.com (C.L.);; 2 Nanjing Anji Biotechnology Co., Ltd., Nanjing 210033, China; 3 State Key Laboratory of Natural Medicines, Ministry of Education, China Pharmaceutical University, Nanjing 210009, China

**Keywords:** HCC, micropeptides, MP60, RBM10, EMT

## Abstract

**Background:** Micropeptides, encoded by non-coding RNAs, play a pivotal role in various cellular functions. While several micropeptides have already been linked to HCC, their roles remain incompletely understood. Our study identifies MP60, a conserved micropeptide strongly associated with HCC progression. **Methods and Results:** By analyzing The Cancer Genome Atlas (TCGA) dataset, we assessed the coding potential of long non-coding RNAs (lncRNAs) with significant expression changes in HCC. Our findings reveal that ENST0000614292, a transcript of *LINC01138*, exhibited the highest coding potential, encoding a putative 60-amino-acid micropeptide, which we have named MP60 and confirmed the expression of MP60 in HCC tissues, with a nuclear localization. MP60 directly interacts with RNA-binding motif protein 10 (RBM10) and downregulates its expression. Additionally, MP60 modulates EMT. Functional analyses demonstrated that MP60 promotes cellular proliferation and migration, while reducing cellular adhesion, translated by enhanced tumorigenesis in vivo. Notably, MP60 expression is markedly increased in HCC tissues and is associated with a poorer prognosis. **Conclusions:** These findings identify MP60 as a potential biomarker and therapeutic target in HCC, linking its oncogenic effects to EMT modulation and tumor progression.

## Simple Summary

Hepatocellular carcinoma (HCC) remains a major global health challenge due to its aggressive behavior and limited therapeutic options. In this study, we identified a micropeptide, called MP60, encoded by a genomic region previously considered non-coding. MP60 was found to be highly expressed in HCC tissues and associated with poorer clinical outcomes. Mechanistically, MP60 interacts with RBM10 and represses its expression. Furthermore, MP60 promotes epithelial–mesenchymal transition (EMT) in HCC. Our findings suggest that MP60 may serve as both a prognostic biomarker and a potential therapeutic target in HCC.

## 1. Introduction

Hepatocellular carcinoma (HCC) represents a global public health burden, characterized by rapid tumor growth, elevated metastasis potential, treatment resistance, and frequent recurrence [1,2]. These challenges underscore the urgent necessity to explore the molecular mechanisms driving HCC progression and to identify novel diagnostic biomarkers and therapeutic targets, with specific focusing on long non-coding RNAs (lncRNAs) and micropeptides [3,4].

Micropeptides are a class of novel functional molecules, typically fewer than 100 amino acids in length, that are translated from small open reading frames (sORFs) within endogenous non-coding RNAs (ncRNAs) [5]. Several HCC-associated peptides have been discovered, although some lack rigorous validation regarding their endogenous expression. For example, KRASIM, a 99-amino-acid peptide, inhibits the growth of HCC stem cells by modulating KRAS signaling [6]. In 2019, Guo et al. [7] identified a 57-amino acid micropeptide encoded by *lncRNA-ZFAS1* that is significantly upregulated in HCC tissues and promotes tumor progression through increased intracellular reactive oxygen species. Furthermore, SMIM30, a 59-amino-acid peptide, interacts with SRC/YES1, enhancing HCC cell growth [8]. PINT87aa, initially identified in glioblastoma and consisting of 87 amino acids, has also been detected in HCC, where it triggers cellular senescence through mitochondrial autophagy [9]. Another micropeptide, MPM, located on the inner mitochondrial membrane and consisting of 56 amino acids, suppresses HCC activity by inhibiting mitochondrial respiration [10]. Other notable HCC-related peptides include LINC013026-68AA (68 amino acids) [11], STMP1 (47 amino acids) [12], AC115619-22AA (22 amino acids) [13].

Our team has recently discovered a novel micropeptide that marks the fifth identified micropeptide promoting HCC. Previous studies have identified the lncRNA *LINC01138* as a prognostic marker in HCC, that elevated expression levels are associated with unfavorable patient outcomes [14,15,16,17,18]. Specifically, *LINC01138* transcripts (NR_027468), which are non-coding and do not yield micropeptides, have been implicated in HCC progression due to their interaction with protein arginine methyltransferase 5 (PRMT5) [19]. In contrast, our study reveals a distinct cross-species transcript of *LINC01138* (ENST00000614292), conserved in both humans and mice, which encodes a 60-amino acid micropeptide—designated MP60.

Functional characterization shows that MP60 directly binds RNA-binding motif protein 10 (RBM10), a critical regulator of alternative splicing [20], influencing various cellular processes, including cell proliferation [21,22], apoptosis [23,24,25,26], adhesion [27,28], and cytoskeletal organization [27]. Dysregulation of RBM10 has been implicated in several of diseases, notably cancer such as lung, breast, and ovarian, besides neurological disorders and specific genetic syndromes. This wide array of associations underscores the profound clinical significance of RBM10. Furthermore, its regulatory mechanisms encompass X-inactivation, auto-regulation, and post-translational modifications, highlighting the complexity and versatility of its biological functions [21]. In HCC, RBM10 suppresses tumor progression by downregulating EGFR and phosphorylated ERK, thereby promoting apoptosis [29].

Here, we report that MP60 interacts with and downregulates RBM10. Additionally, MP60 modulates EMT and promotes HCC progression.

## 2. Results

### 2.1. HCC-Associated LINC01138 (ENST00000614292) Is Conserved in Humans and Mice and Shows Potential for Encoding Micropeptides

**Figure 1:**
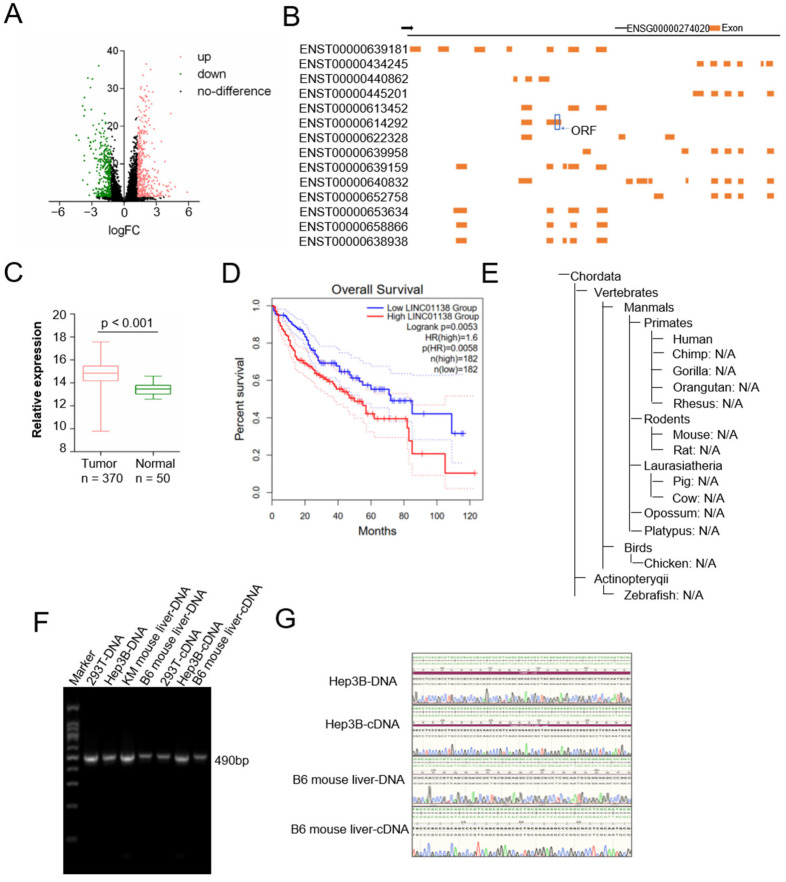
Cross-species conservation of *LINC01138* and its encoded micropeptide MP60. (**A**) Volcano plot illustrating the differential expression of genes in HCC samples compared to normal samples from the TCGA database. (**B**) Schematic representation of 14 distinct transcripts of *LINC01138*, with boxes indicating ORFs with potential coding capacity. (**C**) Relative expression of LINC01138 in HCC samples from the TCGA database. (**D**) Kaplan-Meier analysis of the correlation between *LINC01138* levels and overall survival in HCC samples. (**E**) Conservation analysis of *LINC01138* (ENST0000614292) retrieved from the NONCODE database. (**F**) Expression levels of *LINC01138* (ENST0000614292) detected via PCR in various cell types (293T, Hep3B, KM mouse liver, and B6 mouse liver). (**G**) Sanger sequencing results confirming the conservation of *LINC01138* (ENST0000614292) in Hep3B cells and mouse liver tissues. The uncropped bolts are shown in Supplementary Materials.

A meticulous analysis of the Cancer Genome Atlas (TCGA) database on HCC identified 444 differentially expressed lncRNAs from 370 HCC samples and 50 normal tissue samples, using stringent thresholds (log|fold change| > 1.25, *p*-value ≤ 0.05), as depicted in Figure 1A. These lncRNAs are responsible for generating 1178 distinct transcripts listed in the Ensembl database (Version 106). Advanced bioinformatic tools like MiPepid [30], CPAT [31], CPC2 [32], and PhyloCSF [33] pinpointed *LINC01138* (ENST00000614292) as the lncRNA with the utmost coding potential, as detailed in Table 1. This particular lncRNA possesses the ability to encode a novel 60 amino acids peptide, MP60. *LINC01138*, mapped to the chromosomal location 1q21.2 and documented in the Ensembl genome browser (Version 106), expresses 14 unique transcripts.

The open reading frame (ORF) encoding MP60 resides within a non-overlapping segment of ENST000000614292, distinct from other transcripts (Figure 1B). Notably, the ORF of this transcript is highly expressed in HCC and is significantly associated with adverse clinical outcomes. (Figure 1C,D). Prior research, based on the NONCODE database [34] had suggested that ENST000000614292 is documented only in the human genome (Figure 1F). However, our recent findings, supported by PCR amplification and Sanger sequencing, affirm its consistent presence in both human and mouse genomes (Figure 1E,G). This suggests that MP60, encoded by *LINC01138*, may be a functional peptide.

### 2.2. LINC01138 (ENST00000614292) Encodes a 60-Amino-Acid Micropeptide (MP60) Localized in the Nucleus

**Figure 2:**
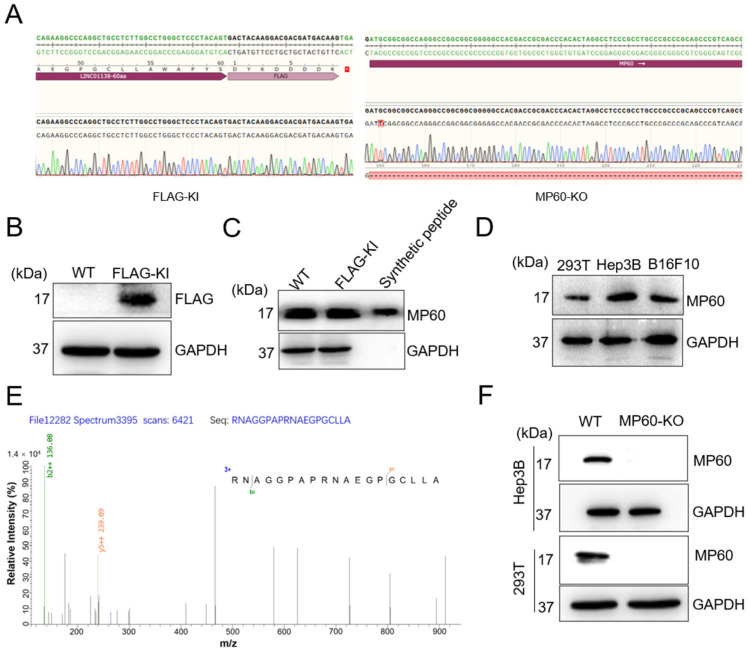
*LINC01138* (ENST0000614292) encodes a 60-amino-acid micropeptide (MP60). (**A**) The Sanger sequencing results showed that Flag Tag is inserted in the MP60 locus in 293T cells. (**B**) MP60 ORF is capable of initiating translation and can be detected by immunoblotting with the Flag Tag antibody. (**C**) Endogenous expression and synthesis of MP60 are verified by immunoblotting with an antibody specific to MP60. (**D**) Endogenous expression of MP60 in 293T, Hep3B and B16F10 cells are detected by immunoblotting with antibody against MP60. (**E**) LC/MS-MS spectra of micropeptide MP60 from the 293T-Flag-KI cell lysate. (**F**) Endogenous expression of MP60 is detected by Western blot with antibody against MP60 in wild type (WT) or MP60 knock out (MP60-KO) Hep3B and 293T cells. Representative images stained with indicated antibodies from three independent experiments. The original images of the Western blotting figures can be found in Supplementary Materials.

To investigate the micropeptide-encoding potential of *LINC01138*, CRISPR/Cas9-mediated homologous recombination was used to insert a Flag tag upstream of the MP60 stop codon in 293T cells. The knock-in success was verified via PCR genotyping and Sanger sequencing (Figure 2A). Western blot analysis further corroborated the presence of MP60 as a Flag-tagged fusion protein in the modified 293T-Flag-KI cells (Figure 2B). Additionally, a rabbit polyclonal antibody targeting MP60 was generated, exhibiting high title and specificity. This antibody effectively identified both endogenous and synthetic MP60 (Figure 2C) and detected MP60 expression in 293T, Hep3B, and B16F10 cells (Figure 2D). These findings underscore the widespread expression of MP60 across various cell types. Immunoprecipitation coupled with mass spectrometry (LC-MS/MS) provided further evidence for the existence of MP60 in 293T-Flag-KI cells (Figure 2E). Western blot analysis also revealed that MP60 expression was absent in cells lacking the ORF, specifically in 293T and Hep3B cells (Figure 2F). Collectively, these findings strongly support that *LINC01138* encodes a stable 60-amino-acid micropeptide expressed across both human and mouse cells.

**Figure 3:**
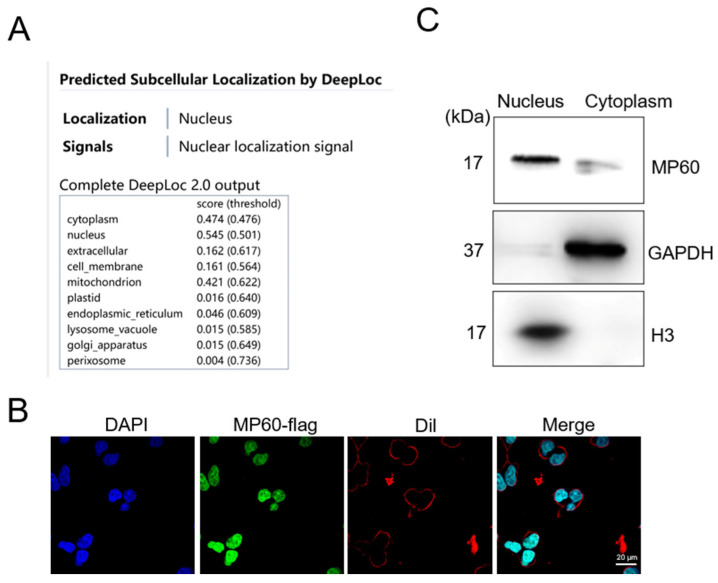
MP60 is localized to the nucleus. (**A**) MP60 subcellular localization was predicted by DeepLoc 2.0 (https://services.healthtech.dtu.dk/services/DeepLoc-2.0/ accessed on 23 November 2023.). (**B**) Confocal microscopy to visualize the subcellular localization of MP60 in 293T-Flag-KI cells. Scale bars, 20 μm. (**C**) The expression of MP60 in nucleus are detected by immunoblotting with antibody against MP60. Representative images stained with indicated antibodies from three independent experiments. The original images of the Western blotting figures can be found in Supplementary Materials.

Given that subcellular localization is pivotal for their function. Utilizing DeepLoc 2.0 [35], a tool that predicts protein localization from sequence features, we determined that MP60 is likely localized in the nucleus (Figure 3A). This prediction was subsequently validated through laser confocal microscopy (Figure 3B) and further supported by cellular fractionation followed by Western blot analysis (Figure 3C).

### 2.3. High Expression of MP60 in HCC Promotes Tumor Growth and Metastasis, and Predicts Poor Prognosis

**Figure 4:**
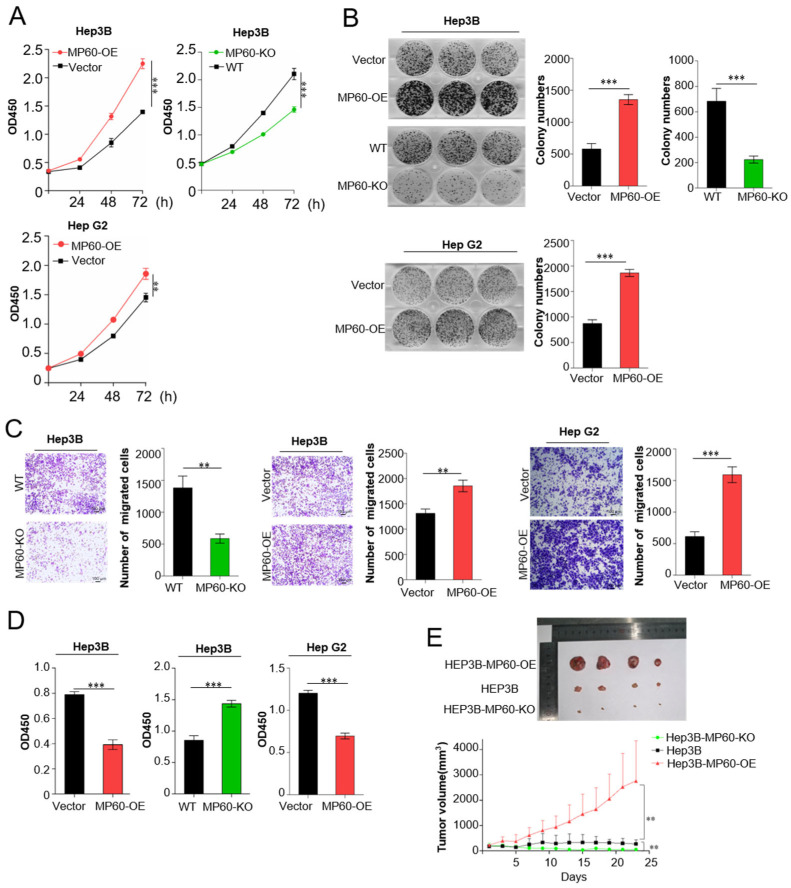
Role of MP60 in HCC Development. The tumor-promoting effect of MP60 on HCC is reflected in cell proliferation (**A**) (n = 5), clone formation assay (**B**) (n = 3), cell migration (**C**) (n = 3), cell adhesion assay (**D**) (n = 5) and nude mice tumor progression model (**E**) (n = 4). Scale bars, 100 μm. Data are reported as the mean ± s.d. Specific n values of biologically independent experiments. *t*-test, ** *p*  <  0.01, *** *p*  <  0.001.

To evaluate the role of MP60 in HCC, a malignancy characterized by dysregulated RBM10, we performed knockout (MP60-KO) and overexpression (MP60-OE) studies in HCC cell lines. Cell viability assays (Figure 4A) and colony formation assays (Figure 4B) demonstrated that overexpressing MP60 led to increased proliferation in both Hep3B and Hep G2 cells. Transwell chambers, migration assays (Figure 4C) further showed that elevated MP60 levels promoted HCC cell migration. Additionally, adhesion experiments (Figure 4D) indicated that higher MP60 concentrations reduced the adhesion capacity of HCC cells. in addition, in vivo xenograft tumor model in nude mice showed that MP60 promoted HCC tumor progression and tumor cells over-expressing MP60 significantly proliferate rapidly after tumor implementation (Figure 4E). Conversely, knocking out MP60 significantly hindered the proliferation, migration, and adhesion of Hep3B cells, collectively confirming MP60′s oncogenic role in HCC.

By analyzing HCC data from the TCGA database, we observed upregulated MP60 expression in HCC samples (Figure 1C). Kaplan–Meier survival analysis (Figure 1D) further revealed that patients with high MP60 expression had markedly poorer overall survival than those with low expression. These findings underscore MP60′s critical involvement in HCC progression, correlating with unfavorable outcomes, and suggesting its potential as a prognostic biomarker for the disease.

### 2.4. MP60 Binds Directly to RBM10 and Modulates Its Expression

**Figure 5:**
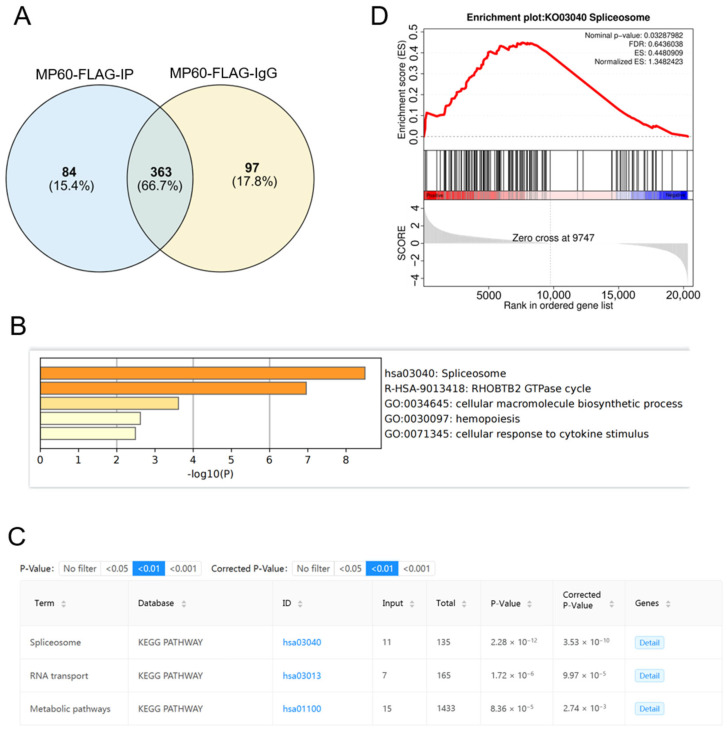
MP60 interacts with RBM10. (**A**) Venn diagram illustrating the number of enriched binding proteins associated with peptide MP60 identified by Co-IP and LC-MS/MS. (**B**) GO analysis results for the MP60-binding proteins were obtained from Co-IP and LC-MS/MS. (**C**) The KEGG analysis results for the MP60-binding proteins were derived from Co-IP and LC-MS/MS. (**D**) RNA-Seq data from MP60-KO Hep3B cells indicated an association with the spliceosome, as determined by GSEA-KEGG enrichment analysis.

Given the limited sequence homology of MP60 with known proteins, we investigated its potential protein–protein interactions. Co-immunoprecipitation (Co-IP) followed by mass spectrometry analysis was employed to investigate these interactions. Initially, using an anti-Flag antibody, we co-immunoprecipitated lysates from 293T-Flag-KI cells and subsequent MS analysis quantified the proteins enriched in the precipitated complex, identifying 84 distinct candidates. (Figure 5A). Kyoto Encyclopedia of Genes and Genomes (KEGG) and Gene Ontology (GO) analyses indicated a significant enrichment in the spliceosome signaling pathway (Figure 5B,C, Table 2). Notably, RBM10 emerged as the principal protein in this pathway. Furthermore, Gene Set Enrichment Analysis (GSEA) of RNA sequencing data from MP60-KO Hep3B cells showed a strong correlation with spliceosomes (Figure 5D).

**Figure 6:**
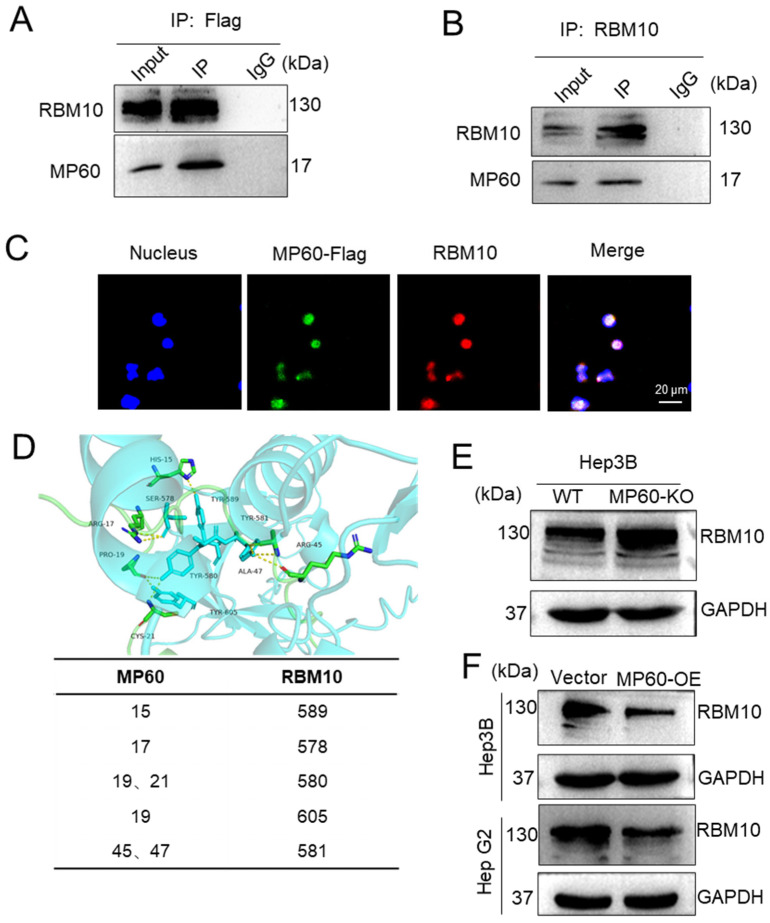
MP60 regulates RBM10 expression. Immunoblot confirmed the interaction of RBM10 and MP60 following immunoprecipitation with anti-Flag antibodies (**A**) and anti-RBM10 antibodies (**B**). (**C**) Confocal microscopy to visualize the subcellular localization of MP60 and RBM10 in 293T-Flag-KI cells, Scale bars, 20 μm. (**D**) Molecular docking results of MP60 and RBM10. (**E**) Western blot of RBM10 in Hep3B cells after MP60 knockout. (**F**) Western blot of RBM10 in Hep3B cells and Hep G2 cells after MP60 overexpression. (**A**,**B**,**E**,**F**) Representative image staining with indicated antibodies from three independent experiments is shown. The original images of the Western blotting figures can be found in Supplementary Materials.

Subsequent Co-IP experiments confirmed the interaction between RBM10 and MP60 (Figure 6A,B). RBM10, a core spliceosomal protein involved in pri-mRNA splicing, is primarily localized in the nucleus. Laser confocal microscopy further supported this interaction by demonstrating the co-localization of MP60 and RBM10 within the nucleus (Figure 6C). Additionally, structural modeling performed with AlphaFold3 predicted potential interaction sites between MP60 and RBM10, supporting the possibility of their direct binding (Figure 6D).

Previous studies have identified RBM10 as a tumor suppressor gene in various cancers, including lung, liver, breast, pancreatic, and colorectal cancers [36]. Given this background, we investigated the effect of MP60 on RBM10 expression. Hep3B cells lacking MP60 exhibited a significant upregulation of RBM10 expression compared to control cells (Figure 6E). Conversely, Hep3B and Hep G2 cells overexpressing MP60 showed a considerable downregulation of RBM10 (Figure 6F). Collectively, these findings suggest that MP60 interacts with RBM10 and acts as an inhibitor of its expression.

### 2.5. MP60 Regulates EMT in HCC

**Figure 7:**
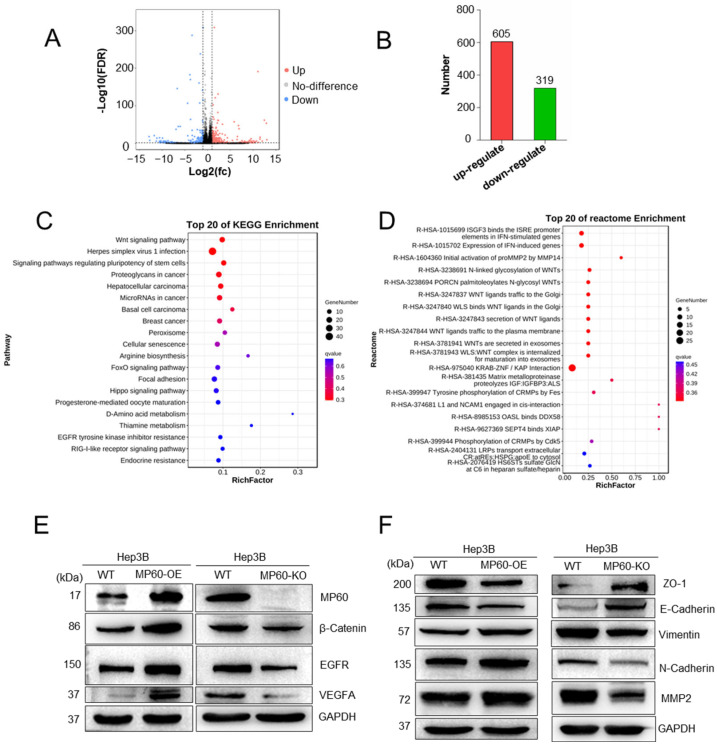
MP60 regulates EMT in HCC cells. (**A**) Volcano plot of differential gene expression from RNA-Seq in MP60-KO Hep3B cells. (**B**) Number of up-regulated and down-regulated genes in MP60-KO Hep3B cells. (**C**) The KEGG enrichment of RNA-seq analysis of MP60 KO. (**D**) The reactome enrichment of RNA-seq analysis of MP60 KO. (**E**) Expression changes in β-Catenin, EGFR, and VEGFA in Hep3B with MP60-KO or MP60-OE, cells. (**F**) Overexpression of MP60 results in the downregulation of epithelial markers (ZO-1, E-Cadherin) and the upregulation of mesenchymal markers (Vimentin, N-Cadherin, MMP2) in Hep3B cells. Knockout of MP60 enhances the expression of epithelial markers (ZO-1, E-Cadherin) and reduces mesenchymal markers (Vimentin, N-Cadherin, MMP2) in Hep3B. (**E**,**F**) Representative image staining with indicated antibodies from three independent experiments is shown. The original images of the Western blotting figures can be found in Supplementary Materials.

MP60 expression is associated with poor prognosis and promotes tumor progression in HCC. To investigate the mechanistic underpinnings of MP60, we performed RNA sequencing (RNA-Seq) on CRISPR/Cas9-generated MP60-KO Hep3B cells. Comparative transcriptomic analysis between MP60-KO and wild-type cells identified 605 significantly upregulated and 319 downregulated genes (∣log_2_FC∣ > 1.0, adjusted *p*-value ≤ 0.05; Figure 7A,B), demonstrating substantial transcriptomic remodeling upon MP60 loss.

Enrichment analyses (KEGG and Reactome) revealed that differentially expressed genes were enriched in cancer-related pathways, including Wnt/β-catenin signaling, angiogenesis, focal adhesion, and EGFR signaling (Figure 7C,D). Consistent with these findings, Western blot analysis showed that MP60 overexpression increased the protein levels of β-Catenin, EGFR, and VEGFA (Figure 7E), whereas MP60 knockdown reduced their expression, further supporting MP60′s role in regulating these pathways.

Given the established involvement of these pathways in EMT, we examined the expression of key EMT markers. MP60 overexpression induced a mesenchymal phenotype, characterized by upregulation of N-cadherin, vimentin, and MMP2, and downregulation of E-cadherin and ZO-1 (Figure 7F). Conversely, MP60 knockout attenuated EMT, resulting in opposite changes in these markers (Figure 7F). Together, these data demonstrate that MP60 promotes EMT in HCC cells.

Previous studies have shown that RBM10, an RNA-binding motif protein and tumor suppressor, inhibits HCC progression by downregulating EGFR and phosphorylated ERK [37]. Cao et al. [38] further reported that RBM10 modulates EMT through β-Catenin in lung cancer. Our finding that MP60 suppresses RBM10 expression while influencing both EGFR and β-Catenin levels suggests a potential functional interaction between MP60 and RBM10 in EMT regulation in HCC. Future work is necessary to elucidate the precise mechanism underlying this interaction and its implications for HCC pathogenesis.

## 3. Discussion

The advent of ribosome profiling, proteomics, and predictive algorithms has revolutionized our comprehension of the molecular biology landscape, enabling the establishment of extensive micropeptide databases [39,40,41]. Despite these advancements, the field still confronts challenges in verifying the accuracy of these predictions, validating the expression capabilities of micropeptides, and elucidating their functions and underlying mechanisms. While previous studies have classified certain *LINC01138* transcripts, including NR_027468 and NR_104014, as non-coding [19], our findings provide compelling evidence that a distinct transcript, ENST00000614292, conserved in both humans and mice, encodes a 60-amino acid micropeptide. This unique transcript contains a distinct ORF that does not overlap with ORFs of other transcripts, highlighting the imperative to individually evaluate the coding potential of each lncRNA transcript.

Our findings contribute to the evolving understanding that distinct transcripts of the same non-coding RNA can exert diverse functions in HCC. While certain LINC01138 transcripts have been implicated in HCC progression through interactions with PRMT5 [19], our study uncovers a novel micropeptide encoded by *LINC01138* (ENST0000614292). This discovery highlights the importance of identifying and characterizing previously unrecognized novel micropeptides in HCC. Notably, our RNA-Seq analysis suggests that MP60 influences spliceosome activity, which may have implications in herpes virus infection (Figure 7C). This observation opens up new avenues for exploring the role of MP60 in disease pathogenesis and therapeutic targeting. Similarly, whether different transcripts of *LINC01138* (and their translated micropeptides) exhibit synergistic or antagonistic effects in tumorigenesis represents an intriguing and worthwhile question for future investigations.

**Figure 8:**
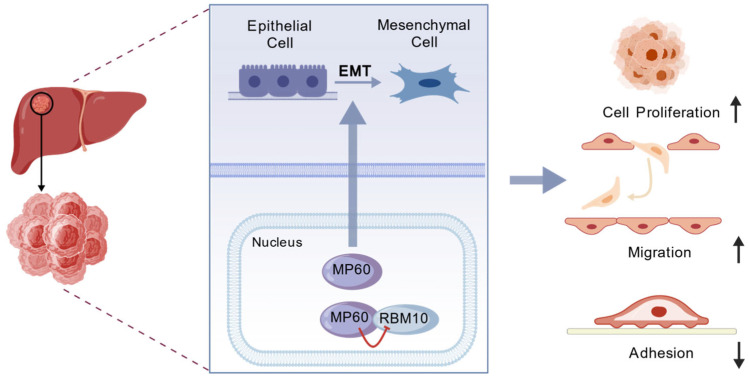
The proposed model for the role of LINC01138-encoded MP60 in HCC. Upward arrows represent promotion, and downward arrows represent inhibition.

RBM10, a critical RNA-binding protein and splicing regulator, modulates the expression of a wide array of cellular proteins and has been implicated in multiple pathological processes such as cancer and viral infections. Although recent studies have shed light on certain regulatory mechanisms involving RBM10—including alternative splicing, autoregulation, and functional interplay with RBM5—the comprehensive network of upstream controllers and downstream effectors remains poorly characterized [42]. In this study, we demonstrate that MP60 interacts with and suppresses the expression of RBM10, thereby uncovering a previously unrecognized signaling axis that modulates RBM10 expression (Figure 8). This finding provides new mechanistic insights into the regulatory landscape of RBM10 and enhances our understanding of its role in cellular processes, particularly in spliceosome assembly and function, as well as in disease contexts. We will further investigate the regulatory relationships between MP60 and RBM10 and between β-catenin and EMT, as well as the connections of MP60 with the spliceosome, immunity, and angiogenesis. Although this study identified the interaction between MP60 and RBM10 and predicted their binding sites using AlphaFold3, the precise mode of interaction remains to be experimentally validated. Furthermore, as MP60 is a newly identified micropeptide, its structural features have not yet been investigated. Therefore, structural characterization of MP60 and detailed mechanistic studies of its interaction with RBM10 will be among our primary research priorities in the future.

EMT is a pivotal process in tumorigenesis and cancer progression, characterized by the loss of epithelial markers and acquisition of mesenchymal traits, which collectively facilitate metastasis and aggressive tumor behavior. Our results demonstrate that MP60 upregulation enhances EMT in HCC cells and significantly accelerates tumor progression in vivo, indicating that MP60 plays a critical role in promoting invasive and metastatic phenotypes in HCC (Figure 8). Furthermore, we found that MP60 suppresses the expression of RBM10, a known regulator of EMT via the β-catenin signaling pathway. These findings collectively suggest a novel MP60-RBM10 regulatory axis that potentially modulates EMT in HCC. Future studies will focus on elucidating the precise mechanistic interactions between MP60 and RBM10 and their collective impact on EMT, which may provide new insights into therapeutic strategies targeting metastasis in HCC. Moving forward, we will employ a broader range of tumor cell types and models to further investigate the potential of MP60 in the diagnosis and treatment of HCC, and explore whether MP60 could serve as a novel therapeutic target for HCC.

## 4. Conclusions

In summary, our study unveils a novel MP60-RBM10 regulatory axis contributing to HCC progression. The identification of MP60 as a functional micropeptide encoded by a specific *LINC01138* transcript expands our understanding of the coding potential of lncRNAs and their implications in cancer biology. Future efforts to elucidate the mechanisms governing the MP60-RBM10 interaction and its downstream effects on HCC pathogenesis could pave the way for innovative therapeutic strategies targeting this pathway. Furthermore, our RNA-Seq data implicate MP60 in diverse processes including spliceosome function, immunity, angiogenesis, and herpesvirus infection, presenting compelling avenues for future investigation.

## 5. Materials and Methods

### 5.1. TCGA Database Analysis

RNA sequencing data for 370 HCC samples and 50 adjacent normal liver tissues were obtained from TCGA data portal (https://portal.gdc.cancer.gov/ accessed on 20 October 2020). Raw read counts were normalized using the Reads Per Kilobase Million (RPKM) method, accounting for both library size and transcript length. This normalization ensured a fair comparison of gene expression levels across different samples. To assess the prognostic significance of *LINC01138* expression, Kaplan-Meier survival curves were generated. Patients were stratified into groups based on *LINC01138* expression levels (high vs. low). The Kaplan-Meier method was used to estimate the survival probability over time for each group, and the curves were compared using the log-rank test to determine statistically significant differences in survival between the groups.

### 5.2. Bioinformatic Prediction

MiPepid (https://github.com/MindAI/MiPepid accessed on 11 March 2022), CPAT (https://cpat.readthedocs.io/en/latest/ accessed on 11 March 2022), and CPC2 (https://github.com/gao-lab/CPC2_standalone accessed on 14 March 2022) were installed locally. All human transcripts from the Ensembl database (Version 106) were downloaded for prediction. Results were ranked in descending order based on MiPepid predictions. PhyloCSF (https://genome.ucsc.edu/ accessed on 21 March 2022) and TransLnc (http://bio-bigdata.hrbmu.edu.cn/TransLnc/ accessed on 25 March 2022) were used to further validate coding potential.

MP60 nucleotide sequence:

ATGCGGCGGCCAGGGCCGGCGGCGGGGGCCACGACCGCGACCCACACTAGGCCTCCCGCCTGCCCGCCCGCAGCCCGTCAGCGGAGCGCTGCGGGAGGCCCGGCGCCTCGCAATGCGGGAGGCCCGGCGCCTCGCAATGCAGAAGGCCCAGGCTGCCTCTTGGCCTGGGCTCCCTACAGTTGA

### 5.3. Cell Lines and Cell Culture

The human HCC cell lines Hep3B and Hep G2, and the human embryonic kidney cell line HEK293T were obtained from the American Type Culture Collection (ATCC). Hep3B and Hep G2 cells were maintained in RPMI-1640 medium (Life Technologies, Carlsbad, CA, USA) supplemented with 10% fetal bovine serum (FBS) (Bioind, Cromwell, CT, USA). HEK293T cells were cultured in Dulbecco’s Modified Eagle Medium (DMEM) (Life Technologies, Carlsbad, CA, USA) supplemented with 10% FBS. All cell lines were incubated at 37 °C in a humidified atmosphere containing 5% CO_2_.

### 5.4. Cell Transfection and Vector Construction

Lentiviral vectors (pLenti-CMV-GFP-Puro, General Biol, Chuzhou, China) containing the cDNA sequence of interest were co-transfected with packaging plasmids psPAX2 (General Biol, Chuzhou, China) and PMD2.G (General Biol, Chuzhou, China) into HEK293T cells using Lipofectamine 3000 (Invitrogen, Carlsbad, CA, USA) according to the manufacturer’s instructions. Stable cell lines expressing the target gene were generated by infecting cells with the lentiviral particles and subsequently selecting for puromycin resistance.

### 5.5. Gene Editing

The designed sgRNA was inserted into the PX458 plasmid (General Biol, Chuzhou, China), and single-stranded donor DNA (ssODN) was designed based on the Knock-in sequence characteristics. The plasmid and donor DNA were co-transfected into cells. Monoclonal screening was performed, and genomic DNA from monoclonal cells was used as a template for PCR. The PCR products were sequenced to confirm successful genome editing.

For Flag Knock-in at MP60:

sgRNA: GCCTGGGCCACACTCAACTGTAGG

Donor DNA: CAATGCAGAAGGCCCAGGCTGCCTCTTGGCCTGGGCTCCCTACAGTGACTACAAGGACGACGATGACAAGTGAGTGTGGCCCAGGCCAACCGAACACTAGAGCAGCAGCGATGGAAGGCT

For MP60 knockout:

sgRNA1: TCCACATCTATGTTCATGATGCGG

sgRNA2: GCCTGGGCCACACTCAACTGTAGG

Primers for PCR:

MP60-F: CCCTGAACAGCAAGACCAATA

MP60-R: AACCTGGCCTAAGTTGATAACC

Donor DNA and primers were commercially synthesized (General Biol, Chuzhou, China).

### 5.6. RNA Isolation and RT-qPCR Analysis

Total RNA was isolated using TRIZOL reagent (Invitrogen, Carlsbad, CA, USA) according to the manufacturer’s instructions. And cDNA was synthesized using HiScript III RT SuperMix for qPCR (R323-01, Vazyme, Nanjing, China) following the manufacturer’s protocol. The mRNA levels were detected by ChamQ SYBR qPCR Master Mix (Q311-02, Vazyme, Nanjing, China) and performed on ABI QuantStudio 3 Real-Time PCR System (Applied Biosystems, Carlsbad, CA, USA). The mRNA expression was normalized by the expression of GAPDH and relative expression levels were calculated using the 2^−ΔΔCT^ method in cell.

Primers for RT-qPCR:

MP60-F: GACCCACACTAGGCCTCCC

MP60-R: TAGGGAGCCCAGGCCAAG

GAPDH-F: GGTGTGAACCATGAGAAGTATGA

GAPDH-R: GAGTCCTTCCACGATACCAAAG

Primers were commercially synthesized (General Biol, Chuzhou, China).

### 5.7. Protein Extraction and Western Blot Analysis

Total protein was extracted from cell lysates using RIPA lysis buffer (Beyotime Biotechnology, Shanghai, China) supplemented with Halt Protease Inhibitor Cocktail (Thermo Fisher Scientific, Waltham, MA, USA). Protein concentrations were determined using the bicinchoninic acid (BCA) assay. Equal amounts of protein were separated by sodium dodecyl sulfate-polyacrylamide gel electrophoresis (SDS-PAGE) and transferred onto a polyvinylidene difluoride (PVDF) membrane (EMD Millipore, Billerica, MA, USA).

The membrane was blocked with 5% non-fat milk in Tris-buffered saline with Tween-20 (TBST) for 2 h at room temperature and then incubated overnight at 4 °C with the following primary antibodies:

Flag (Abcam, ab205606, Cambridge, MA, USA), MP60 (1:1000), EGFR (Proteintech, 18986-1-AP, Wuhan, China), VEGFA (Proteintech, 82864-16-RR, Wuhan, China), E-cadherin (Proteintech, 20874-1-AP, Wuhan, China), N-cadherin (Proteintech, 22018-1-AP, Wuhan, China), ZO-1 (Cell Signaling Technology, 5406, Danvers, MA, USA), Vimentin (Abcam, ab92547 Cambridge, MA, USA), MMP2 (Abcam, ab92536 Cambridge, MA, USA), GAPDH (Proteintech, 10494-1-AP, Wuhan, China), Histone H3 (Proteintech, 17168-1-AP, Wuhan, China), β-Catenin(Cell Signaling Technology, 9562, Danvers, MA, USA). After washing with TBST, the membrane was incubated with horseradish peroxidase (HRP)-conjugated secondary antibodies for 1 h at room temperature. Protein bands were visualized using a Western Blot Detection kit (Beyotime Biotechnology, Shanghai, China) and the TANON-5200 system (Tanon, Shanghai, China).

### 5.8. Co-Immunoprecipitation (Co-IP)

Cell lysates were prepared using IP Lysis Buffer (Beyotime Biotechnology, Shanghai, China) supplemented with Halt Protease Inhibitor Cocktail (Thermo Fisher Scientific, Waltham, MA, USA). The lysates were incubated with the primary antibody of interest overnight at 4 °C. The following day, the antibody-bound protein complex was incubated with 40 μL of Protein A Agarose Beads (Cell Signaling Technology, 9863, Danvers, MA, USA) for 2 h at 4 °C. After three washes with Wash Buffer (0.5 M Tris-HCl pH 7.4, 1.5 M NaCl), the protein-bound beads were resuspended in 5x loading buffer (Yeasen, Shanghai, China) and boiled for 10 min at 95 °C. The samples were then stored at −20 °C or immediately analyzed by Western blotting.

### 5.9. Cell Proliferation Assay

Cell proliferation was assessed using the Cell Counting Kit-8 (CCK-8) assay (Hanbio, Shanghai, China). Briefly, cells were seeded in 96-well plates at a density of 1 × 10^4^ cells per well in 100 μL of complete medium. After 48 h of incubation, CCK-8 reagent was added to each well, and the plates were kept for 1 h at 37 °C. Absorbance at 450 nm was measured using a Multiskan Plate Reader (Thermo Fisher Scientific, Waltham, MA, USA) at the indicated time points.

### 5.10. Transwell Migration Assays

The HCC cells were harvested and seeded with serum-free medium into the upper chambers at 4 × 10^4^ cells/well, and the bottom chambers were filled with culture media and 10% FBS as a chemoattractant, and then incubated for 48 h at 37 °C, successfully translocated cells were fixed by ice-cold methanol and stained with 2% crystal violet, and imaged using an inverted microscope (Olympus, X53DP74, Tokyo, Japan).

### 5.11. Adhesion Assay

The bottom of a 96-well plate was coated with Matrigel (Corning Incorporated, Corning, NY, USA). Hep3B (MP60-OE), Hep3B (MP60-KO), Hep G2 (MP60-OE), and control cells were collected and adjusted to an appropriate concentration. The cells were seeded into the Matrigel-coated 96-well plate with five replicate wells per cell type and cultured for 4 h. The supernatant was discarded, and non-adherent cells were washed away. Fresh medium was added to each well, followed by the addition of CCK-8 reagent. After 1.5 h of incubation, the absorbance was measured using a microplate reader, and statistical analysis was performed.

### 5.12. In Vivo Animal Model

Male BALB/c-nude mice (6-8 weeks old) were housed under specific pathogen-free conditions in the animal facility of Nanjing Anji Biotechnology Co., Ltd. All subsequent experimental procedures were conducted in strict accordance with the institutional ethical guidelines for animal research. Briefly, to investigate the impact of MP60 on the tumorigenicity of HCC cells, 1 × 10^7^ Hep3B cells stably overexpressing MP60, MP60 knocked out, or were mock-transfected, were subcutaneously injected into the mice, respectively. The length and width of the tumors in the mice were measured every two days. The tumor volume was calculated using the formula: 0.5 × length × width^2^.

### 5.13. RNA-Seq

Cells were collected and lysed with Trizol reagent (Invitrogen, Carlsbad, CA, USA) for total RNA extraction. After quantification using Nano-1000, Oligo(dT) magnetic beads were used for mRNA enrichment. The enriched mRNA was randomly fragmented into short segments. Double-stranded cDNA was synthesized using the M-MuLV reverse transcriptase system and DNA polymerase I system. Sequencing adapters were added, and cDNA fragments of approximately 200 bp were collected. The final sequencing library was constructed via PCR amplification and subjected to sequencing.

### 5.14. Molecular Docking

The complex structure of the RBM10 protein and micropeptide MP60 was predicted using AlphaFold3 (https://alphafoldserver.com/ accessed on 25 September 2024). The amino acid sequences of RBM10 and MP60 were input into AlphaFold3 to directly predict their complex structure. After obtaining the complex structure, molecular dynamics (MD) simulations were performed using GROMACS software (2024.3). The topology file of the complex was generated using the pdb2gmx command, and a cubic simulation box containing the protein-micropeptide complex was created using the editconf command. The system was solvated with the SPCE water model using the solvate command, and ions were added via the genion command to achieve electrical neutrality. The AMBER99SB force field was employed to ensure accurate description of the protein-micropeptide complex. Energy minimization was performed to eliminate unreasonable conformations, followed by NVT (canonical ensemble) and NPT (isothermal-isobaric ensemble) equilibration simulations to ensure thermal equilibrium. Finally, a formal MD simulation was conducted to observe the structural dynamics of the complex, and RMSD was calculated to evaluate complex stability.

### 5.15. Statistical Analysis

The data are presented as the means ± standard deviation (SD) of a minimum of three biological replicates and were compared using the Student’s *t*-test. Multiple comparisons were made by unpaired, two-tailed Student’s *t*-tests or one-way ANOVA. OS curves were assessed with the Kaplan-Meier method and compared by the Log-rank test. Unless otherwise indicated, statistical tests were conducted using GraphPad Prism (Version 8; La Jolla, CA, USA) software. * *p* < 0.05, ** *p* < 0.01, *** *p* < 0.001.

## Figures and Tables

**Table 1:** Table of predictive coding ability results using multiple methods.

ENST ID	Mipepid	Ribo-Seq	MS	PhyloCSF	m6A	CPAT	CPC2	CNCI
ENST00000614292	0.999996	ribo	1	1	1	0.157888	0.065434	−0.06922
ENST00000333487	0.999996	ribo	0	1	0	0.307358	0.115502	−0.0385
ENST00000435419	0.999993	NA	0	1	0	0.185621	0.066343	−0.06717
ENST00000607286	0.999948	ribo	0	1	1	0.528369	0.081465	−0.04014
ENST00000533481	0.999944	ribo	0	0	1	0.04631	0.025215	−0.01556
ENST00000428940	0.999931	ribo	1	1	1	0.070693	0.138509	−0.06513
ENST00000450728	0.999917	ribo	1	0	0	0.28031	0.286989	−0.04956
ENST00000575741	0.999911	ribo	1	1	1	0.215749	0.5	−0.03482
ENST00000440016	0.999893	NA	0	1	0	0.294139	0.15444	−0.06922
ENST00000498967	0.999885	ribo	0	1	1	0.484077	0.750083	−0.13353

**Table 2:** Table of ranking of spliceosome-related proteins detected by Co-IP.

Protein	Score	Sequence
RMB10	248	4
SRSF4	255	3
SRSF8	139	3
U2AF2	72	2

## Data Availability

All data generated or analyzed in this study are included in this manuscript or in the supplementary files. Original data supporting the results of this study are available from the corresponding author upon reasonable request. The datasets analyzed during the current study are available in the TCGA repository (https://www.cancer.gov/tcga accessed on 20 October 2020).

## Supplementary Materials

The following supporting information can be downloaded at: https://www.mdpi.com/article/10.3390/cancers17172932/s1, The uncropped bolts are shown in Supplementary Materials.
