# Supplementary material for: A Novel Nuclear-Localized Micropeptide, MP60, Promotes Hepatocellular Carcinoma Progression via the Epithelial-Mesenchymal Transition
Source: Cancers (Basel). 2025 Sep 7;17(17):2932. doi: 10.3390/cancers17172932 (PMC12428246; doi:10.3390/cancers17172932)

**1. Figure 1F**

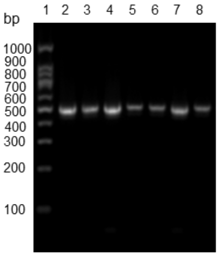


Lane 1: Marker
Lane 2: 293T-DNA
Lane 3: Hep3B-DNA
Lane 4: KM mouse liver-DNA
Lane 5: B6 mouse liver-DNA
Lane 6: 293T-cDNA
Lane 7: Hep3B-cDNA
Lane 8: B6 mouse liver-cDNA

**2. Figure 2B**. Lane 1: 293T cell lysate; Lane 2: MP60-Flag (293T) cell lysate.

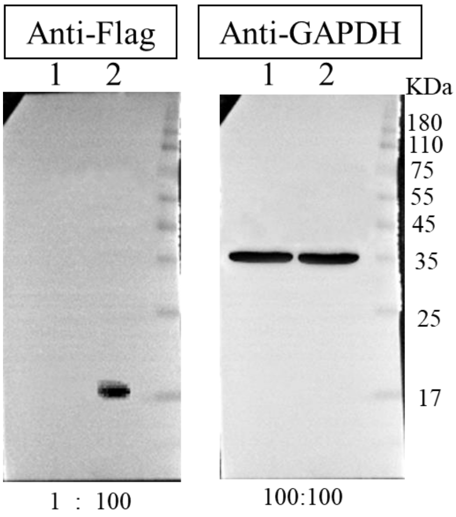


**2.Figure2C**. Lane 1: 293T cell lysate; Lane 2: MP60-Flag (293T) cell lysate; Lane 3: chemically synthesized MP60 peptide.

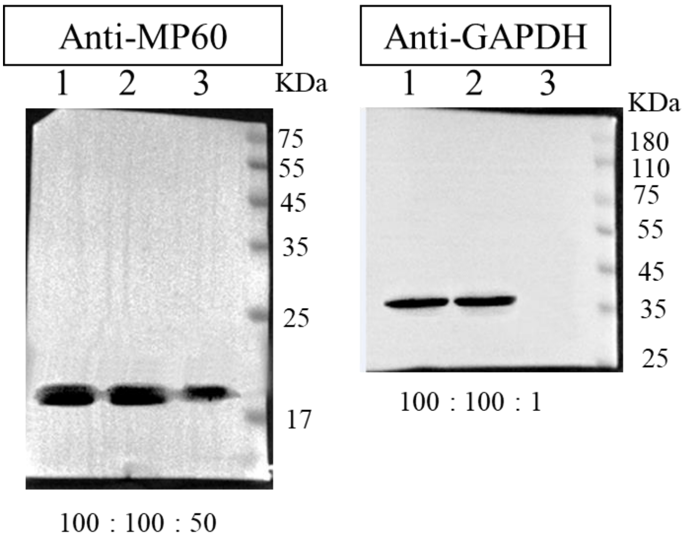

**3.Figure 2D**. Lane 1: 293T cell lysate; Lane 2: Hep3B cell lysate; Lane 3: B16F10 cell lysate.

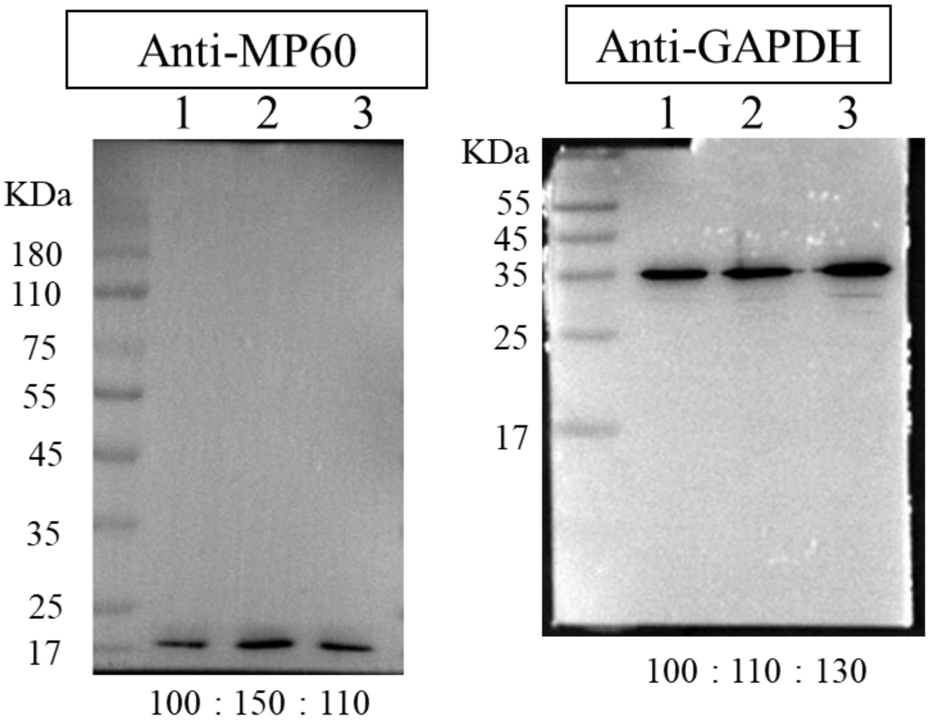


**4.Figure2F**. Lane 1: Hep3B cell lysate; Lane 2: MP60-KO (Hep3B) cell lysate; Lane 3: 293T cell lysate; Lane 4: MP60-KO (293T) cell lysate.

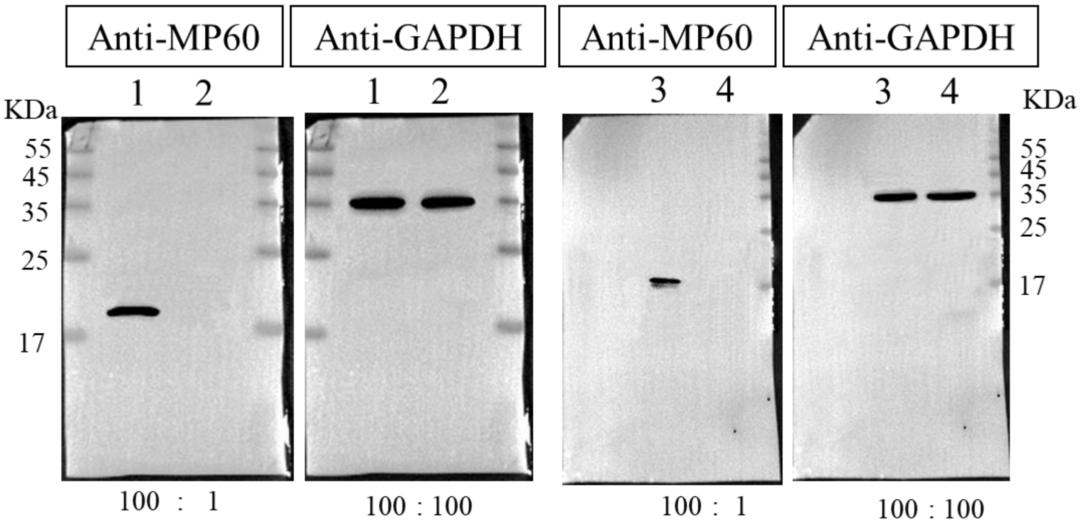

**5.Figure 3C**. Lane 1: 293T nuclear lysate; Lane 2: 293T cytoplasmic lysate.

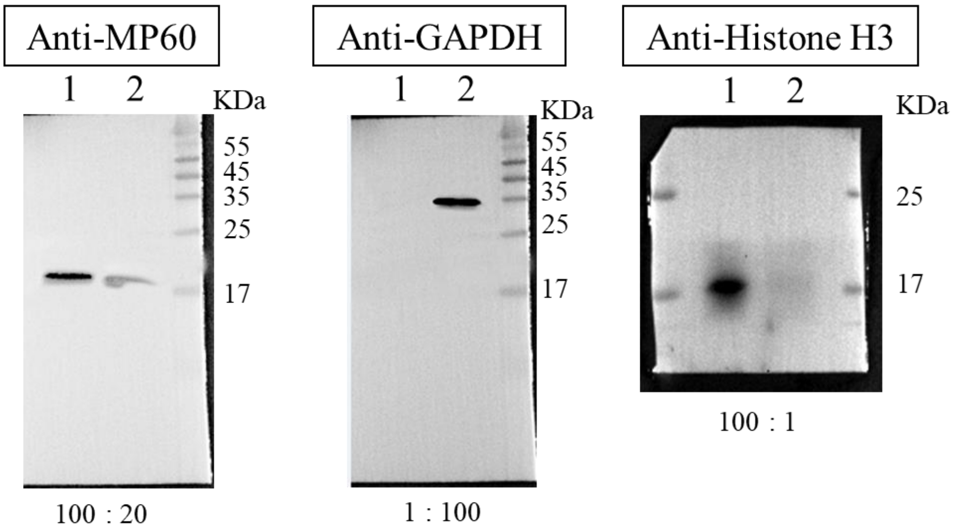


**6.Figure 6A**. Lane 1: 293T cell lysate; Lane 2: Anti-Flag antibody IP product; Lane 3: IgG control.

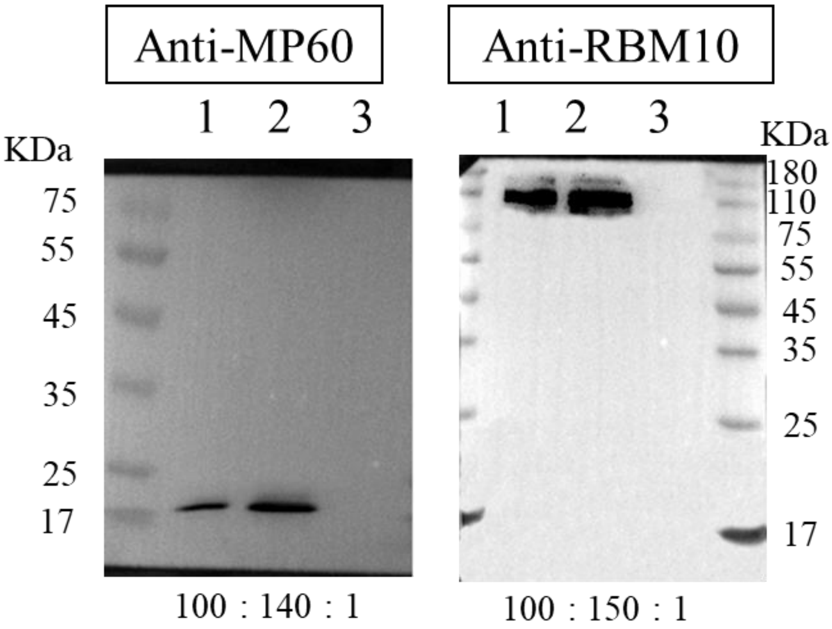

**7.Figure 6B**. Lane 1: 293T cell lysate; Lane 2: Anti-RBM10 antibody IP product; Lane 3: IgG control.

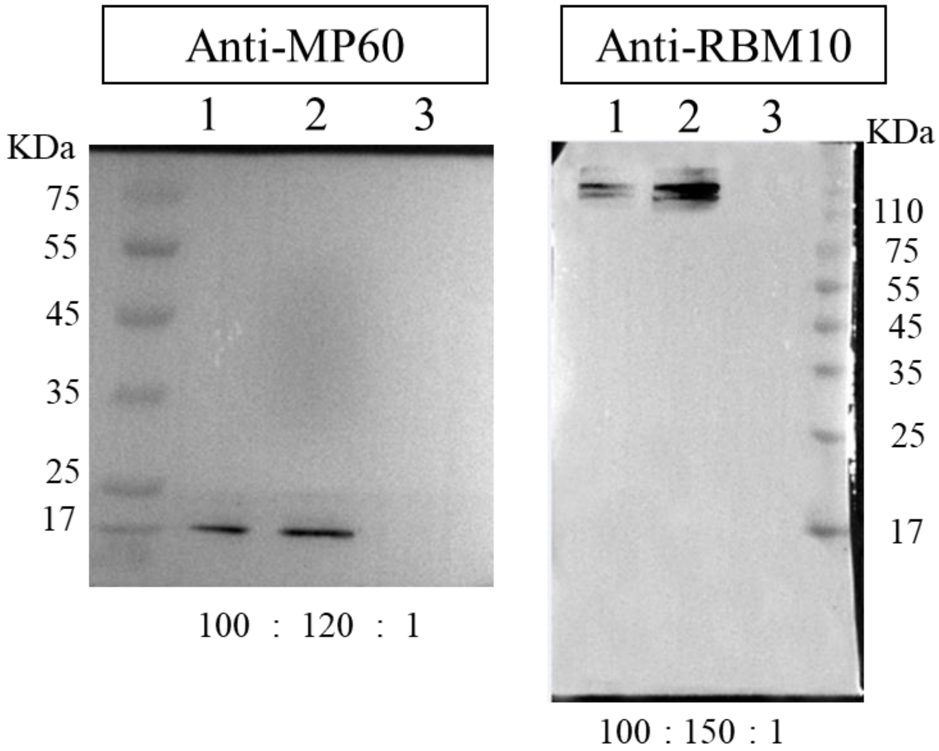


8.**Figure 6E**. Lane 1: Hep3B (Vector) cell lysate; Lane 2: Hep3B (MP60-KO) cell lysate.

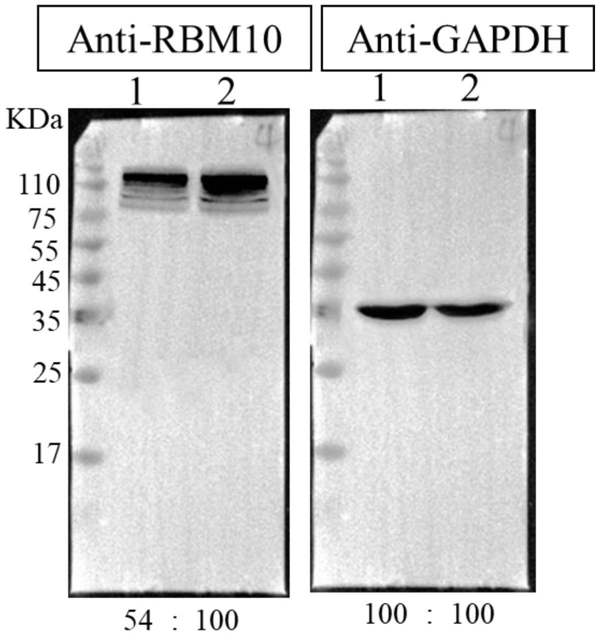

**9.Figure 6F**. Lane 1: Hep3B (Vector) cell lysate; Lane 2: Hep3B (MP60-OE) cell lysate; Lane 3: HepG2 (Vector) cell lysate; Lane 4: HepG2 (MP60-OE) cell lysate.

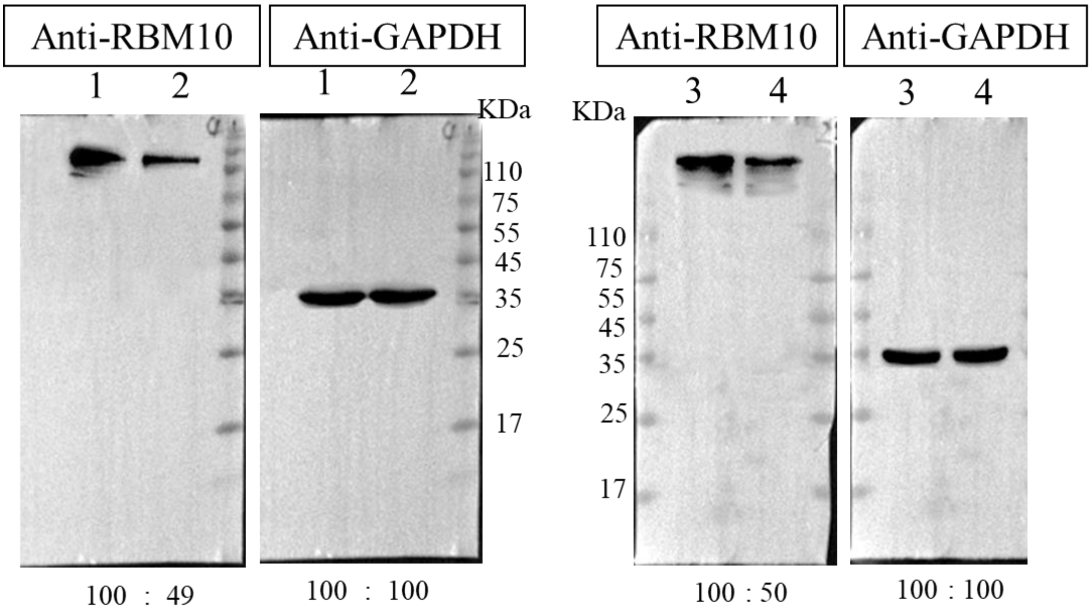


**10.Figure 7E.** Lane 1: Hep3B (Vector) cell lysate; Lane 2: Hep3B (MP60-OE) cell lysate; Lane 3: HepG2 (Vector) cell lysate; Lane 4: HepG2 (MP60-OE) cell lysate.

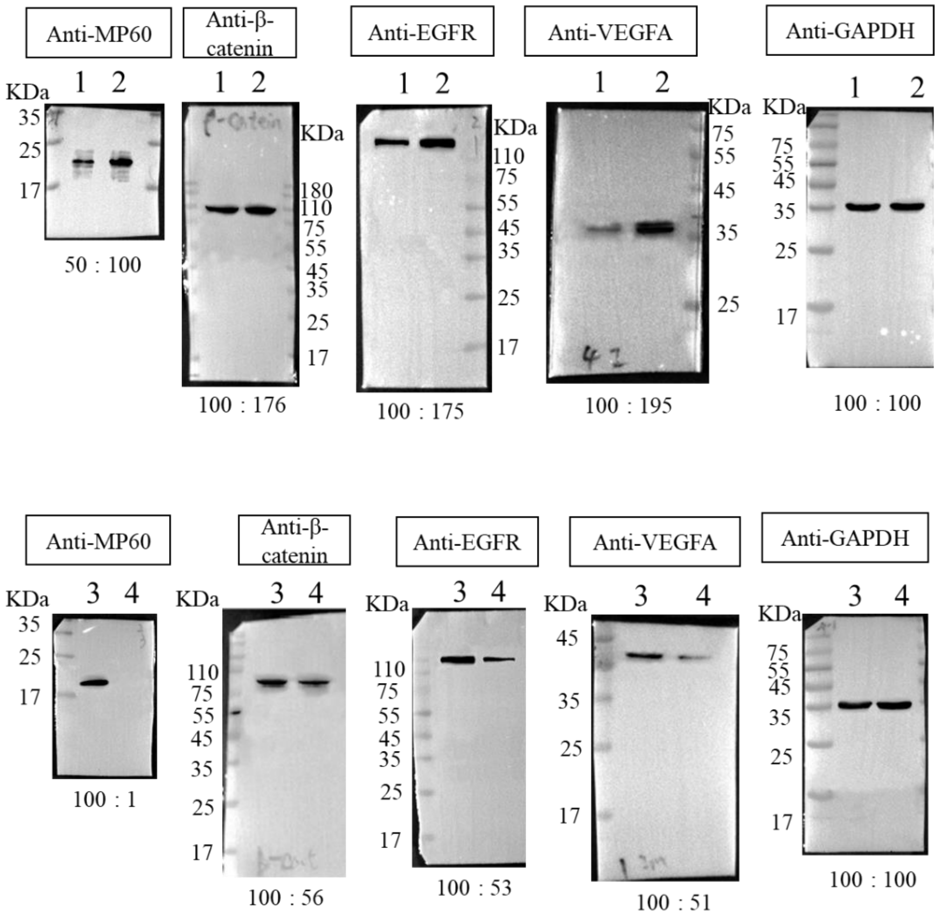

**11.figure 7F**. Lane 1: Hep3B cell lysate; Lane 2: Hep3B (MP60-OE) cell lysate.

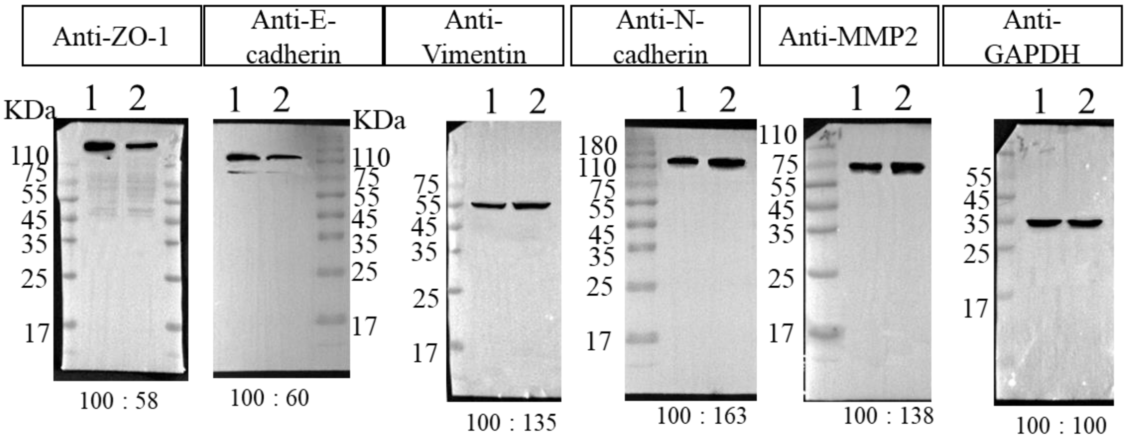


**12.Figure 7F**. Lane 1: Hep3B cell lysate; Lane 2: Hep3B (MP60-KO) cell lysate.

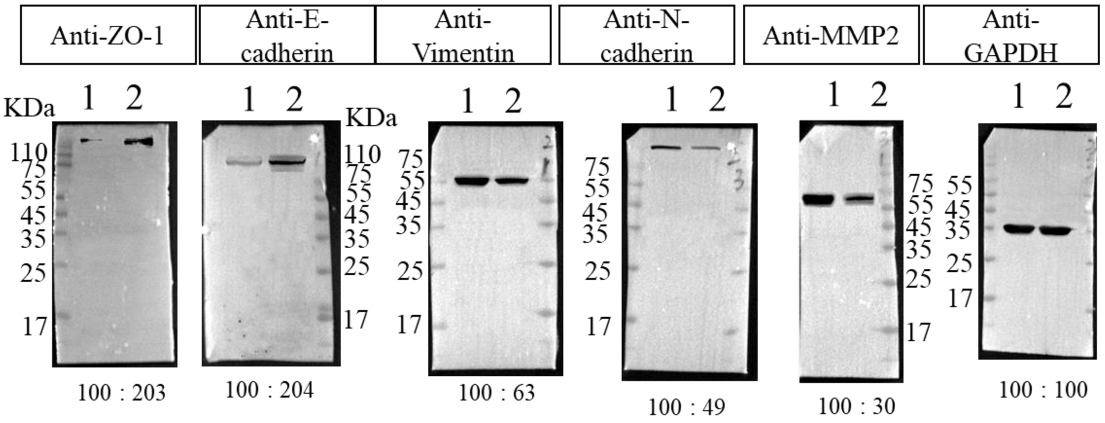

Supplement: Supplementary file 1 [file cancers-17-02932-s001.zip › cancers-3845868-supplementary.pdf]
